# Starving honey bee (*Apis mellifera*) larvae signal pheromonally to worker bees

**DOI:** 10.1038/srep22359

**Published:** 2016-02-29

**Authors:** Xu Jiang He, Xue Chuan Zhang, Wu Jun Jiang, Andrew B. Barron, Jian Hui Zhang, Zhi Jiang Zeng

**Affiliations:** 1 Honeybee Research Institute, Jiangxi Agricultural University, Nanchang, 330045, China; 2 Biomarker Technologies Co., Ltd. Beijing, 101300, China; 3 Department of Biological Sciences, Macquarie University, North Ryde, NSW 2109, Australia

## Abstract

Cooperative brood care is diagnostic of animal societies. This is particularly true for the advanced social insects, and the honey bee is the best understood of the insect societies. A brood pheromone signaling the presence of larvae in a bee colony has been characterised and well studied, but here we explored whether honey bee larvae actively signal their food needs pheromonally to workers. We show that starving honey bee larvae signal to workers via increased production of the volatile pheromone E-*β*-ocimene. Analysis of volatile pheromones produced by food-deprived and fed larvae with gas chromatography-mass spectrometry showed that starving larvae produced more E-*β*-ocimene. Behavioural analyses showed that adding E-*β*-ocimene to empty cells increased the number of worker visits to those cells, and similarly adding E-*β*-ocimene to larvae increased worker visitation rate to the larvae. RNA-seq and qRT-PCR analysis identified 3 genes in the E-*β*-ocimene biosynthetic pathway that were upregulated in larvae following 30 minutes of starvation, and these genes also upregulated in 2-day old larvae compared to 4-day old larvae (2-day old larvae produce the most E-*β*-ocimene). This identifies a pheromonal mechanism by which brood can beg for food from workers to influence the allocation of resources within the colony.

Communication between parents and young offspring for food provisioning presents an area of potential conflict between the amount of food requested by young and the optimal resource allocation by the parents. This has become an area of increasing interest to evolutionary biologists^1^. Recent studies mainly focused on parent-offspring conflicts in mammals and avian species^2^,^3^,^4^. In these species since parents are equally related to their offspring they are expected to favour an even division of food to their young^1^, however, varying condition of young offspring will also influence allocation decisions. In many species parental provision is in relation to begging intensity as a proxy of offspring need and condition rather than the real food needs of hungry sibs^1^. For example, pigeons (*Columba livia*) and sows (*Sus scrofa*) allocated more food to loudspeakers playing begging calls rather than the hungry young^5^,^6^. On the other hand, young offspring often try to acquire a disproportionately larger investment from their parents than their sibs by making louder begging calls and attempt to manipulate their parent to obtain more food than the parents’ optimal resource allocation^3^,^4^. Nevertheless the costs of begging calls curtail call exaggeration and help to ensure their honesty^7^. Furthermore, environmental factors also influence the degree of parent-offspring conflict: for instance, a shortage of food could reduce the honesty of begging signals and increase sibling scramble competition^4^; Adding unrelated broodmates increases barn swallow chicks’ begging intensity^8^. These influential studies of parent-offspring conflict in mammals and birds illustrate the potential complexity of communication over resource allocation.

As an advanced eusocial insect, honey bees have a radically different social organisation for brood rearing. The single queen in the colony lays all the eggs but young larvae are reared by their adult sisters rather than their parents^9^. During the larval stage, each larva is fed 1.5 ± 0.2 times/h by nurse bees not randomly^10^,^11^, and thousands of larvae need to be fed daily^9^. Brood rearing is the primary function of a honey bee colony, but the potential for conflict between brood and nurses over resource allocation in this specialized society has thus far not been explored because it has not been clear whether the largely immobile larvae are capable of begging for food.

Generally, begging signals in vertebrates are body movements and acoustic signals^12^,^13^,^14^,^15^. Pervious studies showed that some insect larvae such as wasp larvae (*Vespa orientalis* F) and beetle larvae (*Nicrophorus vesoilloides*) produce acoustic or tactile signals to beg for food^16^,^17^. It is well documented that larval honey bees produce pheromones capable of altering adult worker behaviour^18^,^19^,^20^. Therefore, it is possible that honey bees could use volatile pheromones as their begging signal to attract workers to them when hungry. This has been demonstrated for bumble bees, although the exact composition of the pheromone remains unknown^21^, and the possibility has been suggested for honey bees^22^. Here we explored whether honey bee larvae signal their hunger pheromonally.

Our findings suggest that a volatile component of brood pheromone, E-*β*-ocimene, is a candidate begging signal for young honey bee larvae. It is actively synthesized by hungry larvae and attracts workers to inspect cells.

## Results

**Figure 1:**
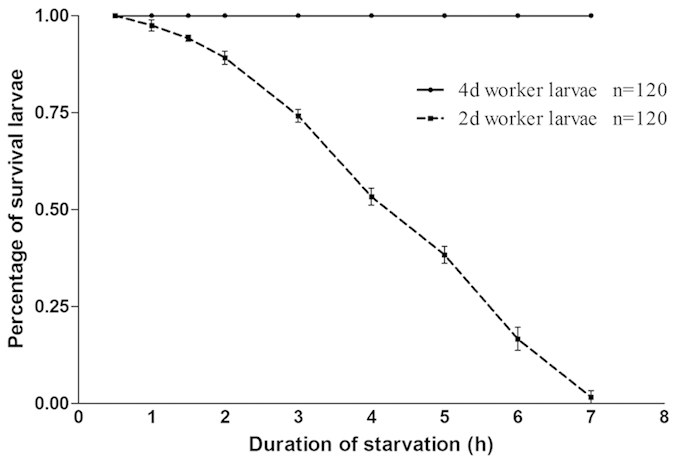
Survival of worker larvae with different durations of starvation from 0 to 7 hours. 4-day old larvae (solid line and diamonds) survived longer than 2-day old larvae (dash line and squares). Forty 2- and 4-day old larvae were tested and the study was replicated three times. The mean + SE of each time point were presented.

Food deprivation affected 2-day old larvae far more severely than 4-day old larvae (Fig. 1). Because of the high mortality observed in 2-day old larvae after more than 2 h starvation, we limited starvation duration to less than this time for all other experiments.

### Experiment 2: NT and GC-MS

**Figure 2:**
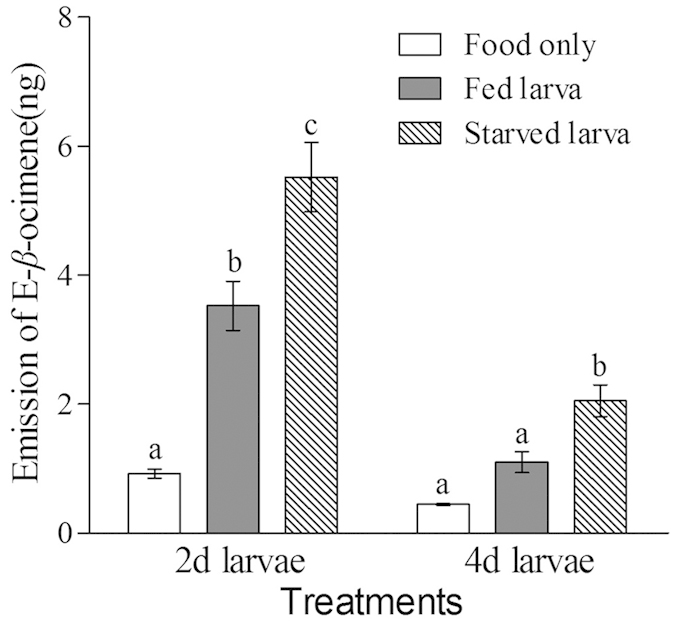
Emission of E-*β*-ocimene from SL (striped bar), FL (gray bar) and food only (open bar). Mean + SE presented in each bar. 2- and 4-day old SL released significantly more E-*β*-ocimene compared to FL (2-day old comparison: n = 6, p = 0.013; 4-day old comparison: n = 6, p = 0.009). Different letters “a”,“b” and “c” on top of bars indicate significant difference (Fisher’s PLSD test, P < 0.05, after ANOVA showed an significant effect), whereas same letter indicates non significant difference (ANOVA P > 0.05).

Nine chemicals were detected in 2- and 4-day worker larva groups: E-*β*-ocimene, Myristic acid, Palmitic acid, Methyl palmitic ester, Stearic acid, Palmitoleic acid, Pentadecanoic acid, Acetic acid and Ethyl acetate. These chemicals mapped to the standard chemicals in the NIST32I database with a relative coefficients >80% for all except Stearic acid which was 78% (Table S1). E-*β*-ocimene was the only chemical which was significantly higher in all starved larvae groups (SL) compared to the respective fed larvae groups (FL) (Fig. 2, Fig. S1), suggesting that it might be the candidate for the larval hunger signal.

### Experiment 3: Effect of E-*β*-ocimene on worker behaviour

**Figure 3:**
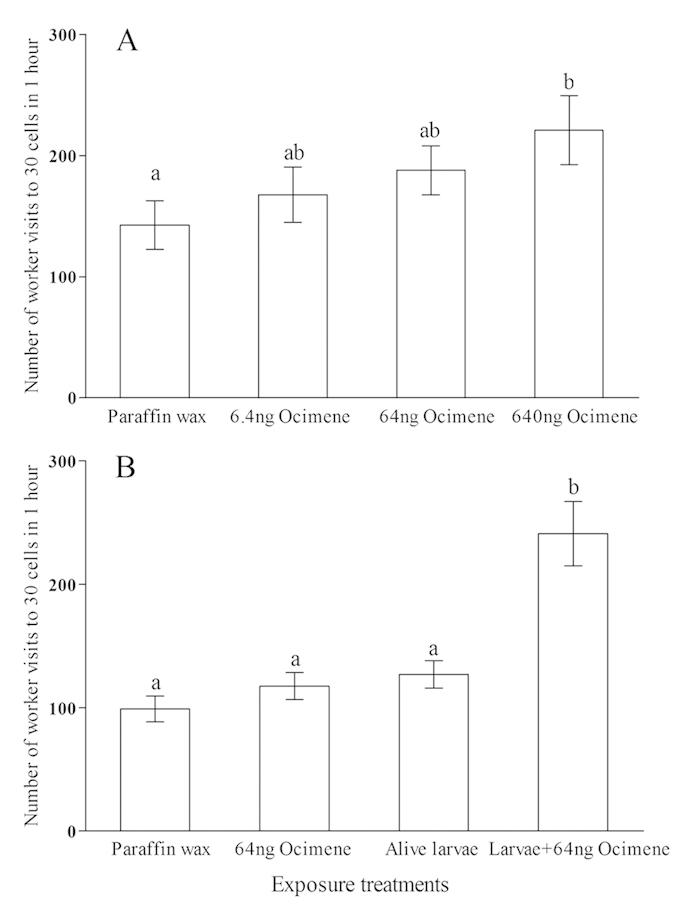
Total number of worker visits to the cells added with (**A**) different amounts of E-*β*-ocimene (Expt 3a) and (**B**) E-*β*-ocimene with or without living larvae (Expt 3b). Mean + SE is presented in each bar. Only the 640 ng E-*β*-ocimene treatment has significantly more visits than the control in Expt 3a (n = 6, p = 0.048), whereas the living larvae supplemented with E-*β*-ocimene has significantly more visits than the larvae, E-*β*-ocimene alone or wax only control groups in Expt 3b (n = 8, p < 0.0001). Different letters “a” and “b” above bars indicate significant differences (P < 0.05) between groups (ANOVA followed by Fisher’s PLSD test).

E-*β*-ocimene increased worker attendance of cells in a dose-dependent manner (Fig. 3A). In addition, workers attended living larvae supplemented with E-*β*-ocimene more than the larvae, E-*β*-ocimene alone or wax only control groups (Fig. 3B).

### Experiment 4: RNA sequence analysis

Four libraries were generated from our experimental groups, and summaries of RNA sequencing analyses are shown in Table S2. In each library, more than 97% clean reads were unique reads of which more than 86% reads were paired reads. Very few clean reads (<2.1%) were multiple mapped reads. Each library had a sufficient coverage of the expected number of distinct genes (stabilized at 3M reads, Table S2). The Pearson correlation coefficient among three biological replicates of each experimental group were all >0.80 (Table S3), which is a conventionally accepted threshold for replicates indicating that there was acceptable sequencing quality and repeatability among the biological replicates of each group^23^.

**Figure 4:**
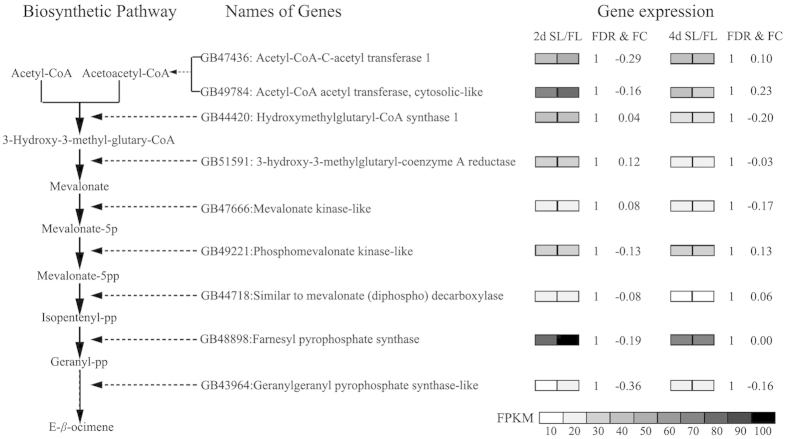
The biosynthetic pathway for E-*β*-ocimene and relative expression of 9 genes involved in different honey bee samples. Honey bee larva *de novo* synthesise E-*β*-ocimene from Acetyl-CoA and Acetoacetyl CoA (left). The name of each gene (middle) and its relative expression (right) in four worker larva groups (day 2 SL, day 2 FL, day 4 SL and day 4 FL) involved in this pathway were presented. The shade of each gene in each larva group indicates its FPKM value (fragments per kilobase of exon per million fragments mapped).

There were only 1 and 12 genes were significantly differentially expressed between SL and FL in 2-day old and 4-day old comparisons respectively (log2 ratio ≥ 2 and FDR < 0.01, Table S4). We noticed that 3 and 5 genes putatively involved in E-*β*-ocimene synthesis were slightly upregulated in SL compared to FL in 2-day old and 4-day old comparisons respectively (Fig. 4).

Many biosynthetic pathways of E-*β*-ocimene have been found in animals and plants (Fig. S2). By mapping to the KEGG pathway database, we found that honey bee larvae had a possible biosynthetic pathway for E-*β*-ocimene involving 9 genes (Fig. 4). It appears that in bees E-*β*-ocimene might be *de novo* synthesised from a Mevalonate pathway (Fig. 4). Furthermore, a transcription factor (Longitudinals lacking protein-like, *llp-like*) involved in the regulation of genes Farnesyl pyrophosphate synthase ( *fps*) and Mevalonate kinase-like (*mk*) could also be involved in the regulation of E-*β*-ocimene production. None of these 9 genes were differentially expressed between SL and FL when starved for 45 min, nor between 2-day old larvae and 4-day old larvae (Fig. 4). Although differences were not significant, three of the nine genes were expressed far more highly in 2-day old larvae than 4-day old larvae (2-day old larvae produce more E-*β*-ocimene). Four of these nine genes [Phosphomevalonate kinase-like (*pk-like*), Geranylgeranyl pyrophosphate synthase-like (*ggps-like*), *fps* and *aatc-like*] were expressed slightly higher in FL compared to SL (Fig. 4).

### Experiment 5: Results of qRT-PCR

**Figure 5:**
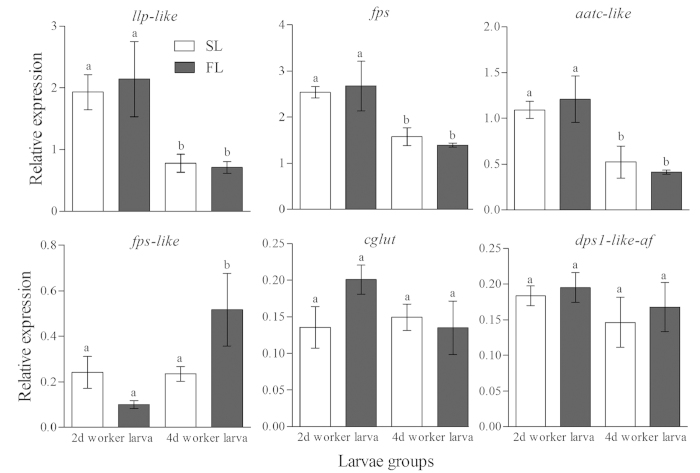
Comparison of relative gene expression level among 2- and 4-day old SL and FL. Mean + SE presented in each bar. Different letters “a” and “b” on top of bars indicate significant differences (P < 0.05, ANOVA followed by Fisher’s PLSD test).

Our RT-PCR analyses focused on the putative genes of the E-*β*-ocimene pathway suggested from Expt 4. None of these genes were significantly differentially expressed between the same age SL and FL that had been starved for 45 minutes (Expt 5a, Fig. 5), which was consistent with the results from Expt 4. However, three of these genes (*llp-like, fps and aatc-like*) were more highly expressed in 2-day old worker larvae compared to 4-day old worker larvae (Fig. 5), which also supported the conclusions of the RNA sequence analyses (Fig. 4). Two-day old larvae produce more E-*β*-ocimene than four-day old larvae (Fig. 2).

**Figure 6:**
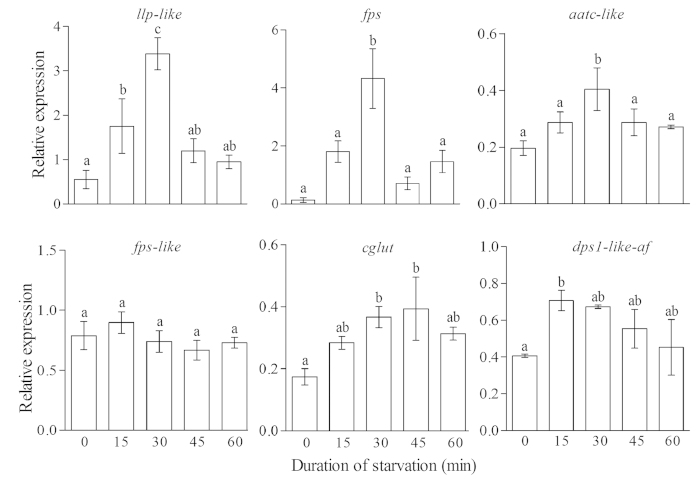
Expression of 6 genes following different durations of starvation from 0 to 60 min in day-2-old worker larvae. Mean + SE was presented in each bar. Different letters “a” and “b” on top of bars indicate significant differences (P < 0.05, ANOVA followed by Fisher’s PLSD test).

We also compared the expression of these nine genes in 2-day old worker larvae that were starved for 0, 15, 30, 45 and 60 min. Three genes were upregulated after 30 min of starvation, and then expression decreased (Fig. 6). The control and other genes showed a different expression pattern (Fig. 6).

## Discussion

In summary, our results have shown that food deprivation increased the amount of the volatile chemical E-*β*-ocimene produced by worker larvae (Fig. 2), and that 2-day old larvae, which are highly susceptible to starvation, produced more E-*β*-ocimene than 4-day old larvae. E-*β*-ocimene increased the number of visits to brood cells by workers (Fig. 3), and hence we propose that E-*β*-ocimene signals the state of larval hunger to workers.

E-*β*-ocimene was first identified from mated honey bee queens^24^ and could promote the acceptance of introduced queens to honey bee colony^25^, and then it was detected from young brood and even brood frames^26^,^27^. A previous study showed that it also accelerates the development of worker hypopharyngeal glands, which are the main glands for secreting royal jelly, and promotes workers to start their foraging earlier^22^. These findings, and our results, are consistent with the interpretation that E-*β*-ocimene signals the need for food by larvae, and that workers react to this pheromonal signal by attending brood cells producing the pheromone, and also over a longer timescale by increasing brood food production and increasing food collection by the colony. We note, however, that while E-*β*-ocimene increased the number of visits workers made to brood cells, we did not observe more food provided to these cells. The amount of food given to a worker larva on a single trip is very small, and it proved to be too difficult to quantify the amount in this study. The decision of how much food to give a larva may be complex and involve more than just the E-*β*-ocimene signal. For example, when inspecting the cells that contain larvae, nurses may find leftover food and this in turn may influence food allocation^11^. Nurses may also gain additional information about larval health and hunger through larval movement^28^. It is possible honey bee larvae may use the volatile E-*β*-ocimene to signal their food need and attract nurses to their cell from a short distance away, but feeding may involve other signals as well. We noted that adding just 64 ng E-*β*-ocimene into a cell containing a living 2-day old worker larva dramatically increased worker visits compared to control treatments (Expt 3b, Fig. 3B). However, adding E-*β*-ocimene alone to a cell was less effective in soliciting a response from workers. Adding 640 ng E-*β*-ocimene diluted in solid paraffin wax to a cell slightly increased workers’ attendance compared to the control (Expt 3a, Fig. 3A). This perhaps suggests the E-*β*-ocimene and other signals from a living larva may have a synergistic effect on the attendance behaviour of worker bees.

RNA-seq results indicated a KEGG pathway in honey bee larvae for *de novo* synthesis of E-*β*-ocimene through the mevalonate pathway. This is similar to the monoterpene synthesis pathway of the bark beetle (*Ips pini*)^29^. Three genes were upregulated in 2-day old larvae compared to four day old larvae (Fig. 5), since two-day old larvae produce significantly more E-*β*-ocimene compared to the four-day olds. Three of the six genes tested from this pathway (Fig. 6) showed elevated expression after 30 minutes of starvation suggesting dynamic genomic regulation of synthesis of E-*β*-ocimene capable of rapid upregulation in response to a starvation stress. However, there was no significant difference between the gene expression of the FL and SL. This is perhaps because the changes in E-*β*-ocimene released from a hungry larva and a fed larva (approximately 2 ng and 1 ng in 2-day old and 4-day old larvae respectively) are infinitesimal and not easily reflected in gene expression. Furthermore, both FL and SL were detected producing E-*β*-ocimene which may have reduced any difference at the gene expression level of analysis. A previous study showed that the highest amount of brood esterifiable pheromones was detected on the anterior part of the larvae, especially high in the salivary gland^30^. As a hydrocarbon and a hunger pheromone, it may be the case that E-*β*-ocimene is produced from (the anterior part around) the mouth area, however, this needs further investigation.

Begging signals have received a great deal of attention because of their possible roles in parent-offspring conflicts over the resourcing of young, but most of this research has been with vertebrate systems. Examples of begging signals from insects are rare. This is no doubt partly because parental care is also generally rare in insects, however sibling care is common in social insects, and obligatory for advanced social insects. Some examples of begging signals have been reported for social insects. Wasp larvae (*Vespa orientalis* F) use their mandibles to rub against the cell wall to make a scraping sound as a hungry signal^16^. Some ant species larvae such as *Myrmica rubra* and *Gnamptogenys stratula* beg for food by swaying behaviour in which they raise their head and neck, and reach and wave towards workers^31^,^32^. The cuticular chemicals from hungry bumblebee (*Bombus terrestris*) and earwig (*Forficula auricularia*) larvae attract greater adult attendance suggesting that these cuticular compounds might serve as a pheromonal signal of hunger^21^,^33^. Our study is the first to chemically identify a pheromonal begging signal in eusocial insects, and to propose an active mechanism whereby larval honey bees signal to workers. While there is no indication of conflict in this signalling system in the present study, it raises the possibility of potential discordance between how much brood signal for food from workers, and how much food is delivered to them, particularly in periods of food shortage for the colony. A previous study showed that hungry larvae were able to attract more inspections and feeding visits than the control group, however, nurses may deposit food only when food quantity within the cell is below a minimum threshold^11^. On the other hand, it is not clear whether brood could use E-*β*-ocimene to manipulate their food allocation. Therefore, studying the possibility for conflict over larval provisioning in honey bees could prove a fascinating test of general theory of parent-offspring conflict which are very different from those of parent-offspring care in most avian or mammalian systems, and provide important information on how food flow is regulated in honey bee colonies.

## Methods and Materials

### Insects

Experiments 1, 2, 4 and 5 used the standard Chinese commercial strain of western honey bee (*Apis mellifera*). Experiments 1, 4 and 5 used bees from three colonies located at the Honeybee Research Institute of Jiangxi Agricultural University (28.46° N, 115.49° E). Experiment 2 used three colonies that were transferred to Nanjing University of Chinese Medicine (32.6° N, 118.56° E). Experiment 3 used bees from five colonies of the standard Australian commercial strain of the western honey bee (mostly *Apis mellifera ligustca*), and was conducted at Macquarie University, New South Wales, Australia (33.76S, 151.11E).

### Experiment 1: sensitivity of larvae of different ages to starvation

We examined the impact of food deprivation on 2- and 4-day old worker larvae to determine the severity of starvation treatments and establish the optimal time points for sampling for pheromone and genomic analyses. Forty 2- and 4-day old larvae were placed in a 20 mL plastic tube without food and kept in an incubator at 34 °C. Larvae were inspected under a microscope to check whether they were breathing and moving at 0.5 h, 1 h, 1.5 h, 2 h, 3 h, 4 h, 5 h, 6 h and 7 h. Larvae that were completely still were assumed to be dead. This study was replicated three times.

### Experiment 2: Measurement of volatile chemicals produced by larvae with needle trap sample collection

A needle trap device system from PAS technology, Magdala, Germany was used to sample honey bee larvae pheromones^34^. Twenty 2-day and ten 4-day old worker larvae and their food were collected from their wax cells. Larvae were placed in cells with abundant food (FL), which had been freshly collected from their own cells, or placed in cells with no food (SL). Each group was immediately put in a 20 mL airtight glass tube and kept in an incubator under 35 °C for 45 min after collection from honey bee hives. The whole sample collection procedure took less than five minutes min per group in a 30 °C air conditioning room.

Sampling needles were used to extract 10 mL gas from the sample tube at 5 mL/min, 20 MPa and 35 °C. The first type of needle preferentially absorbed fatty acid and other oxophile pheromones (fatty acid model needles), and the second preferentially adsorbed non-oxophile pheromones [divinylbenzene (DVB), PDMS and carboxen 1000 (CAR1000) model needles]^34^. In addition to sampling from FL and SL, we also sampled volatiles from cells that contained larval food only as a control group to allow us to distinguish odours from food in our SL and FL samples. Each sample group had six replicates.

For GC-MS analysis two types of chromatographic column (DB-5 and VOC, both were 30 m, 250 μm, 25 μm film thickness, Agilent technolgies) were used for the different needle samples: for the fatty acid needles we used the DB-5 column; for the DVB, PDMS and Carboxen 1000 mixed needles we used the VOC column. The column temperatures were 35 °C for 2 min, then 35 °C to 250 °C at 8 °C /min. then 250 °C for 5 min. The temperature of injection was 250 °C and the pressure of gas helium was 6.7776 psi. Samples were desorbed from extraction needles using 1 mL of helium passed through the needle and into the GC-MS in less than 30 seconds by using a 1 mL sterile syringe (PAS technology). Chemicals identified in GC-MS were then mapped to the NIST32I database.

A preliminary experiment, and previous studies showed that E-*β*-ocimene was a major volatile chemical from honey bee young larvae^22^,^26^,^27^. For calculating the amounts of this pheromone from honey bee samples, we used 1-Nonene (assay ≥ 99.5%, Sigma-Fluka) and E-*β*-ocimene (assay ≥ 90%, Sigma-Aldrich) as internal and external standard substances respectively. Two μL 1-Nonene injections and six levels of E-*β*-ocimene (0 μL, 1 μL, 2 μL, 4 μL, 8 μL and 16 μL) were added into 20 ml ethyl alcohol to establish a standard curve. Afterward, to determine the efficiency of volatile collection with the needles, 2 μL of ethyl alcohol with 1-Nonene (v/v:10000/1) and six levels of E-*β*-ocimene (v/v: 10000/0; 10000/0.5; 10000/1; 10000/2; 10000/4 and 10000/8) were injected into 20 mL airtight glass tubes under 35 °C for 45 min and subsequently the volatiles extracted by needles according to the method above. The correlation coefficients of the two standard curves (DB-5 and VOC columns) were 99.16% and 99.27% respectively suggesting that the NT and GC-MS systems (7890A/5975C, Agilent technolgies Inc., Santa Clara, CA, USA) were stable enough for the later quantitative analysis of these pheromone compounds from samples from honey bee larvae.

For the honey bee samples, 2 μL ethyl alcohol only with 1-Nonene (v/v:10000/1) was injected into the bottom of the airtight glass tube after adding larval samples and the tube was capped immediately afterward for the duration of the starvation treatment.

### Experiment 3: effect of E-*β*-ocimene on worker behaviour

Expt 2 suggested E-*β*-ocimene was produced by hungry larvae. We then examined how worker bees reacted to E-*β*-ocimene in Expt 3a. E-*β*-ocimene was diluted in small paraffin wax (Sigma-Aldrich) pellets 20 ± 1.5 mg that resembled the size of a worker larva. These were added individually to cells of empty wax comb. There were four treatments: wax with 6.4 ng E-*β*-ocimene (which is approximately equal to the E-*β*-ocimene amount detected from a 2-day old worker larva), wax with 64 ng E-*β*-ocimene, wax with 640 ng E-*β*-ocimene and a wax-only control group. For each trial 30 pellets of each type were used: 120 pellets in total. Wax pellets were stuck to the cell bottom so that they could not be easily removed by workers, however a few wax pellets were still cleaned from cells by workers during the assay (Table S5). The frame of comb with these 120 wax pellets was firstly introduced into a bee colony for 10 min to attract workers who climbed onto the frame. The comb frame was then placed in a box along with a frame with 1500 bees, young larvae and a mated queen. The box containing the two frames, approximately 1700–1800 workers and a queen was kept in a dark incubator under 34 °C and the experimental cells containing the wax pellets were observed with an infrared camera. We recorded every incidence when workers placed their heads into cells containing wax pellets for one hour.

In experiment 3b we compared responses of workers to larvae and E-*β*-ocimene. There were four treatments: comb cells contained either: *i*. 64 ng E-*β*-ocimene diluted in a wax pill as above, *ii*. a wax pill with no E-*β*-ocimene, *iii*. a living 2-day old worker larva grafted into the cell, *iv*. living 2-day old worker larvae with a 64 ng E-*β*-ocimene pill. For group *iv*, the wax pill was first stuck to the bottom of the cells, and living larvae were then grafted to lie on the pellets. There were 30 cells per group. The experimental cells were then exposed to bees and observed as for Expt 3a above to record numbers of visits by bees to cells. Very few wax pellets and larvae were removed by workers during the observations (Table S5).

### Experiment 4: RNA-Seq analysis of 2 and 4 day old starved and fed larvae

To examine the effects of food deprivation on larval gene expression 2-day and 4-day old worker larvae were placed in a disinfected plastic tissue culture dish in a biochemical incubator (under 35 °C, 75% humidity) for 45 min, and were then immediately flash-frozen in liquid nitrogen. FL lay on their food collected from their cells for this period. SL had no food. For 2-day old larvae samples, approximately 30 larvae were collected for each RNA sequencing sample, while for 4-day larvae samples 6 were collected. 4-day old larvae are much larger than 2-day old larvae and hence fewer larvae were needed for adequate RNA yield. Three RNA samples from pooled larvae were collected for each experimental group (2-day and 4-day old SL and FL). Within a group each sample was collected from a different colony.

Total RNA of each sample was extracted from the honeybee larvae according to the standard protocol of the TRlzol Reagent (Life technologies, California, USA). RNA integrity and concentration were checked using an Agilent 2100 Bioanalyzer (Agilent Technologies, Inc., Santa Clara, CA, USA).

mRNA was isolated from total RNA using a NEBNext Poly(A) mRNA Magnetic Isolation Module (NEB, E7490). A cDNA library was constructed following the manufacturer’s instructions for the NEBNext Ultra RNA Library Prep Kit (NEB, E7530) and the NEBNext Multiplex Oligos (NEB, E7500) from Illumina. In brief: enriched mRNA was fragmented into approximately 200 nt RNA inserts, which were used as templates to synthesize the cDNA. End-repair/dA-tail and adaptor ligation were then performed on the double-stranded cDNA. Suitable fragments were isolated by Agencourt AMPure XP beads (Beckman Coulter, Inc.), and enriched by PCR amplification. Finally, the constructed cDNA libraries of the honey bee were sequenced on a flow cell using an Illumina HiSeq™ 2500 sequencing platform.

Low quality reads, such as adaptor-only reads or reads with >5% unknown nucleotides were filtered from subsequent analyses. Reads with a sequencing error rate less than 1% (Q20 > 98%) were retained. These remaining clean reads were mapped to the honeybee (*Apis mellifera*) genome (OGSv3.2) using Tophat2 software^35^. The aligned records from the aligners in BAM/SAM format were further examined to remove potential duplicate molecules. Gene expression levels were estimated using FPKM values (fragments per kilobase of exon per million fragments mapped) by the Cufflinks software^36^.

DESeq and Q-value were employed and used to evaluate differential gene expression between starved and fed worker larvae^37^. Gene abundance differences between sample groups were calculated based on the ratio of the FPKM values. The false discovery rate (FDR) control method was used to identify the threshold of the *P*-value in multiple tests in order to compute the significance of the differences. Here, only genes with an absolute value of log2 ratio ≥ 2 and FDR significance score <0.01 were used for subsequent analysis.

Sequences differentially expressed between sample groups were identified by comparison against various protein database by BLASTX, including the National Center for Biotechnology Information (NCBI) non-redundant protein (Nr) database, Swiss-Prot database with a cut-off E-value of 10^−5^. Furthermore, genes were searched against the NCBI non-redundant nucleotide sequence (Nt) database using BLASTn by a cut-off E-value of 10^−5^. Genes were retrieved based on the best BLAST hit (highest score) along with their protein functional annotation. KEGG pathways were assigned to the assembled sequences by Perl script.

### Experiment 5a: Quantitative RT-PCR anlaysis of gene expression differences between starved and fed larvae of different ages

Total RNA from the RNA-Seq samples (Expt 4) were used in qRT-PCR validation of putative differences in gene expression between samples. RNA integrity was determined by agarose gel (1%), electrophoresis, and ethidium bromide staining. The purity (260 nm/280 nm ratio between 1.8 and 2.0 for RNA) and the concentration of each RNA sample were measured in triplicate following methods in the limma function of HTqPCR package of R^38^. RNA samples were standardized to a concentration of 1 μg/μl for reverse transcription. cDNA was synthesized using MLV reverse transcriptase (Takara, Japan) according to the manufacturer’s instructions. GAPDH-1 was used as an internal ‘housekeeping gene’ who’s expression level was similar within all samples and against which levels of expression of our genes of interest were compared^39^,^40^. We focused on six target genes for qRT-PCR analysis (Table S6). Four of these genes were from the E-*β*-ocimene biosynthetic pathway, two control genes unrelated to the E-*β*-ocimene pathway were selected: Ceramide glucosyltransferase (*cglut*) and Decaprenyl-diphosphate synthase subunit 1-like-*Apis florae* (*dps1-like-af*). Primers for these genes were designed using Primer 5.0 software (Table S6).

qRT-PCR cycling conditions were as follows: preliminary 94 °C for 2 min, 40 cycles including 94 °C for 15 sec, 58.9 °C for 30 sec, and 72 °C for 30 sec. The specificity of the PCR products was verified by melt curve analysis for each sample. For each gene, three biological replicates (with five technical replicate for each biological replicate) were performed.

Control and target genes for each sample were run in the same plate to control for interplate variation. The Ct value for each biological replicate was obtained by calculating the mean of three technical replicates. The relative expression level between FL and SL was calculated using the 2−ΔΔCt formula reported by Liu and Saint^41^.

### Experiment 5b: Examination of influence of duration of starvation on gene expression in 2-day old larvae

Three hundred 2-day old worker larvae were sampled from the one honey bee colony. After sampling, 300 larvae were equally divided into five groups: larvae in the first group were immediately flash-frozen in liquid nitrogen without starving as control. The remaining four groups were placed in a plastic tissue culture dish in an incubator (under 35 °C, 75% relative humidity) and deprived of food for different intervals: 15 min, 30 min, 45 min and 60 min. Afterward, samples were immediately flash-frozen in liquid nitrogen. Each sample had three biological replicates taken from the same three honeybee colonies used for Expt 5a. The RNA extraction, qRT-PCR procedures and 6 target genes were the same as for Expt 5a.

### Statistics

All data from GC-MS, behavioural observation and qRT-PCR of each group were analyzed by ANOVA followed by a Fisher’s PLSD test in StatView 5.01 (SAS Institute, Cary, NC, USA).

## Additional Information

**Data availability**: RNA-Seq data of 2-day old starved honey bee worker larva: NCBI SRA: SRS1025814 (ftp://ftp-trace.ncbi.nih.gov/sra/sra-instant/reads/BySample/sra/SRS/SRS102/SRS1025814/). RNA-Seq data of 2-day old fed honey bee worker larva: NCBI SRA: SRS1025817 (ftp://ftp-trace.ncbi.nih.gov/sra/sra-instant/ reads/BySample/sra/SRS/SRS102/SRS1025817/). RNA-Seq data of 4-day old starved honey bee worker larva: NCBI SRA: SRS1025772 (ftp://ftp-trace.ncbi.nih.gov/sra/sra-instant/reads/BySample/sra/SRS/SRS102/ SRS1025772/). RNA-Seq data of 4-day old fed honey bee worker larva: NCBI SRA: SRS1025798 (ftp://ftp-trace. ncbi.nih.gov/sra/sra-instant/reads/BySample/sra/SRS/SRS102/SRS1025798/).

**How to cite this article**: He, X. J. *et al.* Starving honey bee (*Apis mellifera*) larvae signal pheromonally to worker bees. *Sci. Rep.* **6**, 22359; doi: 10.1038/srep22359 (2016).

## Supplementary Material

Supplementary Information
