# Supplementary material for: Starving honey bee (Apis mellifera) larvae signal pheromonally to worker bees
Source: Sci Rep. 2016 Feb 29;6:22359. doi: 10.1038/srep22359 (PMC4770327; doi:10.1038/srep22359)
Supplement: Supplementary Information [file srep22359-s1.pdf]

Manuscript title: Starving honey bee (*Apis mellifera*) larvae signal pheromonally to worker bees

Author list: Xu Jiang He, Xue Chuan Zhang, Wu Jun Jiang, Andrew B. Barron, Jian Hui Zhang and Zhi Jiang Zeng

Table S1 Nine chemicals detected in each treatment by NT and GC-MS

Name		E-β-ocimene (ng)	Palmitic acid (relative amount)	Myristic acid (relative amount)	Methyl palmitic ester (relative amount)	Stearic acid (relative amount)	Palmitoleic aci d (relative amount)	Pentadecanoic acid (relative amount)	Acetic acid (relative amount)	Ethyl acetate (relative amount)
Structure		$C_{10}H_{12}$	$C_{16}H_{32}O_2$	$C_{14}H_{28}O_2$	$C_{17}H_{34}O_2$	$C_{18}H_{36}O_2$	$C_{16}H_{30}O_2$	$C_{15}H_{30}O_2$	$C_2H_4O_2$	$C_4H_8O_2$
Mapping degree to standard chemical		97%	98%	99%	99%	78%	95%	99%	86%	86%
Molar mass		136	256	228	270	284	254	242	60	88
2d worker larvae	Food only	0.922±0.066 a	0.058±0.017 a	0.030±0.018 a	0.041±0.012 a	0.007±0.003 a	0.019±0.008 a	0.005±0.003 a	0.397±0.131 a	0.624±0.081 a
	Fed Larva	3.524±0.380 b	0.112±0.041 a	0.049±0.020 a	0.015±0.005 b	0.020±0.009 a	0.030±0.010 a	0.008±0.005 a	0.630±0.144 b	0.332±0.107 b
	Starved Larva	5.519±0.539 c	0.065±0.025 a	0.047±0.019 a	0.014±0.002 b	0.011±0.006 a	0.025±0.009 a	0.008±0.008 a	0.148±0.044 c	0.074±0.017 c
4d worker larvae	Food only	0.445±0.013 a	0.020±0.002 a	0.011±0.004 a	0.005±0.001 b	0.003±0.000 a	0.006±0.002 a	0.003±0.001 a	0.076±0.026 c	0.082±0.031 c
	Fed Larva	1.100±0.162 a	0.058±0.025 a	0.027±0.009 a	0.006±0.001 b	0.009±0.003 a	0.018±0.005 a	0.009±0.002 a	0.065±0.006 c	0.052±0.018 c
	Starved Larva	2.053±0.247 d	0.204±0.095 a	0.088±0.039 a	0.015±0.007 b	0.021±0.008 a	0.068±0.037 a	0.035±0.019 a	0.041±0.014 c	0.051±0.034 c

Table S1. Nine chemicals detected in each treatment by NT and GC-MS. Amounts of E-*β*-ocimene presented were the absolute values as there were internal and external standards for this compound. Others were quantified as relative amounts dividing by the internal standard, 1-Nonene. Different letters “a” and “b” following the data indicate significant differences (P<0.05, ANOVA followed by Fisher’s PLSD test).

Table S2 Statistics of RNA sequencing

Samples	2d SL-R1		2d SL-R2		2d SL-R3		2d FL-R1		2d FL-R2		2d FL-R3		4d SL-R1		4d SL-R2		4d SL-R3		4d FL-R1		4d FL-R2		4d FL-R3	
Statistical content	Number	Percentage	Number	Percentage	Number	Percentage	Number	Percentage	Number	Percentage	Number	Percentage	Number	Percentage	Number	Percentage	Number	Percentage	Number	Percentage	Number	Percentage	Number	Percentage
Total Reads	33,883,960	100%	34,022,922	100%	32,595,952	100%	33,682,510	100%	32,331,020	100%	31,253,780	100%	35,856,924	100%	32,760,100	100%	39,689,474	100%	32,244,884	100%	32,970,958	100%	36,707,338	100%
mapped Reads	30,153,125	88.99%	30,521,682	89.71%	28,673,275	87.97%	30,270,725	89.87%	28,791,659	89.05%	28,256,968	90.41%	32,735,231	91.29%	29,897,874	91.26%	36,519,851	92.01%	29,366,652	91.07%	30,352,231	92.06%	33,824,241	92.15%
Uniq Map	29,590,276	98.13%	30,011,809	98.33%	28,108,743	98.03%	29,736,953	98.24%	28,194,258	97.93%	27,739,436	98.17%	32,130,421	98.15%	29,344,361	98.15%	35,889,969	98.28%	28,768,273	97.96%	29,853,234	98.36%	33,265,285	98.35%
Multiple Map	562,849	1.87%	509,873	1.67%	564,532	1.97%	533,772	1.76%	597,401	2.07%	517,532	1.83%	604,810	1.85%	553,513	1.85%	629,882	1.72%	598,379	2.04%	498,997	1.64%	558,956	1.65%
Pair Map	26,366,207	87.44%	26,952,369	88.31%	24,759,237	86.35%	26,602,631	87.88%	25,058,796	87.03%	25,145,291	88.99%	29,086,361	88.85%	26,614,234	89.02%	32,507,570	89.01%	25,941,939	88.34%	27,453,235	90.45%	30,394,806	89.86%
Single Map	3,219,249	10.68%	3,015,504	9.88%	3,357,242	11.71%	3,132,749	10.35%	3,199,085	11.11%	2,684,285	9.50%	3,089,592	9.44%	2,817,932	9.43%	3,415,598	9.35%	2,966,228	10.10%	2,446,221	8.06%	2,932,059	8.67%

Table S3 Pearson correlation coefficient among three biological replicates of each treatment

Samples	4d SL-R1	4d SL-R2	4d SL-R3	2d SL-R1	2d SL-R2	2d SL-R3	2d FL-R1	2d FL-R2	2d FL-R3	4d FL-R1	4d FL-R2	4d FL-R3
4d SL-R1	**1**	**0.904662**	**0.9421887**	0.326450839	0.369926066	0.325142989	0.321164486	0.341033665	0.32971777	0.96553721	0.941551317	0.923032939
4d SL-R2	**0.90466204**	**1**	**0.9695872**	0.407290541	0.446772853	0.415839403	0.421836963	0.416057913	0.43401126	0.91116701	0.95847814	0.911857286
4d SL-R3	**0.942188733**	**0.9695872**	**1**	0.433211624	0.483665671	0.449516364	0.450339823	0.444326591	0.47121808	0.93181047	0.980497473	0.954165866
2d SL-R1	0.326450839	0.407290541	0.433211624	**1**	**0.94484766**	**0.958647806**	0.974093836	0.984596925	0.94361191	0.13569222	0.415152643	0.381973919
2d SL-R2	0.369926066	0.446772853	0.483665671	**0.94484766**	**1**	**0.956956034**	0.961534056	0.927510509	0.94406308	0.2359249	0.458244532	0.423658146
2d SL-R3	0.325142989	0.415839403	0.449516364	**0.95864781**	**0.95695603**	**1**	0.981627455	0.942387594	0.97693227	0.15441929	0.421216088	0.384253808
2d FL-R1	0.321164486	0.421836963	0.450339823	0.974093836	0.961534056	0.981627455	**1**	**0.958963678**	**0.9747517**	0.14273733	0.421435813	0.38504482
2d FL-R2	0.341033665	0.416057913	0.444326591	0.984596925	0.927510509	0.942387594	**0.95896368**	**1**	**0.9498053**	0.15677794	0.422977854	0.39146433
2d FL-R3	0.329717771	0.434011263	0.471218083	0.943611914	0.944063079	0.976932267	**0.97475169**	**0.949805284**	**1**	0.16214269	0.442000487	0.409166707
4d FL-R1	0.965537213	0.911167008	0.931810468	0.135692221	0.235924895	0.154419286	0.142737331	0.15677794	0.16214269	**1**	**0.899406**	**0.8624235**
4d FL-R2	0.941551317	0.95847814	0.980497473	0.415152643	0.458244532	0.421216088	0.421435813	0.422977854	0.44200049	**0.899406**	**1**	**0.9821578**
4d FL-R3	0.923032939	0.911857286	0.954165866	0.381973919	0.423658146	0.384253808	0.38504482	0.39146433	0.40916671	**0.8624235**	**0.9821578**	**1**

Table S4 Significantly differentially expressed genes between SL and FL

Comparison	Number of genes	Gene	FDR	log2FC	Regulated	Annotation
2d SL vs FL	1	GB47541	0.0001025	-4.308226	down	PREDICTED: hypothetical protein LOC100578485 [Apis mellifera]
4d SL vs FL	12	GB42046	1.28E-05	1.5518534	up	PREDICTED: transcription factor kayak-like isoform 1 [Apis florea]
		Bee_newGene_1397	0.0005409	3.69528	up	hypothetical protein CAPTEDRAFT_209435 [Capitella teleta]
		GB53865	6.60E-06	1.7057078	up	PREDICTED: hypothetical protein LOC413596 [Apis mellifera]
		GB48917	0.0001832	1.1800141	up	PREDICTED: hypothetical protein LOC100577268 [Apis mellifera]
		GB47493	8.89E-07	1.4890718	up	homeobox protein H17 [Apis mellifera]
		GB45040	0.0001386	-1.419029	down	PREDICTED: uncharacterized protein LOC100869404 [Apis florea]
		GB54178	0.0046727	1.263793	up	PREDICTED: pyroglutamylated RFamide peptide receptor-like [Apis florea]
		GB49390	0.0012957	1.185596	up	PREDICTED: receptor-type tyrosine-protein phosphatase R [Apis mellifera]
		GB54634	0.0046727	1.7147185	up	PREDICTED: hypothetical protein LOC725260 [Apis mellifera]
		GB50526	0.0098727	1.1059127	up	PREDICTED: sodium-coupled monocarboxylate transporter 2-like [Apis mellifera]
		GB47399	1.24E-06	1.4526621	up	PREDICTED: hypothetical protein LOC724412 [Apis mellifera]
		GB47924	0.0014005	-1.074448	down	PREDICTED: hypothetical protein LOC100577028 [Apis mellifera]

Table S5 Larvae and wax pills were cleanned of each treatment in behavioural experiments

Experiments	Treatments	Items were cleanned	Number of larvae or wax were cleanned							
			Replicate1	Replicate2	Replicate3	Replicate4	Replicate5	Replicate6	Replicate7	Replicate8
Experiment 3a	Alive larvae	Larva	2	0	1	0	0	0	0	0
	Larvae+64ng ocimene	Larva	7	6	7	3	0	7	6	3
	Wax pills	Wax	1	0	0	0	0	0	0	1
	64ng ocimene	Wax	0	0	0	0	1	0	0	0
	Larvae+64ng ocimene	Wax	1	1	0	0	1	0	0	1
Experiment 3b	Wax	Wax	0	2	0	0	0	0	/	/
	6.4ng ocimene	Wax	4	1	0	0	0	0	/	/
	64ng ocimene	Wax	4	2	0	1	0	0	/	/
	640ng ocimene	Wax	5	1	0	1	0	0	/	/

Table S6 Genes and corresponding primers used in qRT-PCR

Genes	Primer	Annealing Temperature
GAPDH-1	5'GAGATGATGACCCTTTTGGC 3'<br>5'GGTGAAGACGCCAGTGGACTC 3'	58.9℃
fps	5' CGTTCTCGCTATGCGTTTCG 3'<br>5' GTTCAGCTGTAGCTCGTTGC 3'	61.3℃
llp-like	5'AATCCGTCCAAACATCCCATCA 3'<br>5' CACCTGGTGCTTCTGCCAAT 3'	62.5℃
cglut	5' CAATTGACGAGGTCGGTGGA 3'<br>5' CAGGAAGCACAGGCACCTAA 3'	56.9℃
fps-like	5' GCTGCCAACGGACCAACTACTC 3'<br>5' CACCCACACGACCAAATCTACG 3'	59.4℃
aatc-like	5' AAGTTGGCGTGGAACCAAGA 3'<br>5'TCCTAGCACCAGATGCACCA 3'	61.3℃
dps1-like-af	5' GAGCATCAACCACTTCAGAGACA3'<br>5' AGTTATTTACATTCGCACCCAA 3'	59.4℃

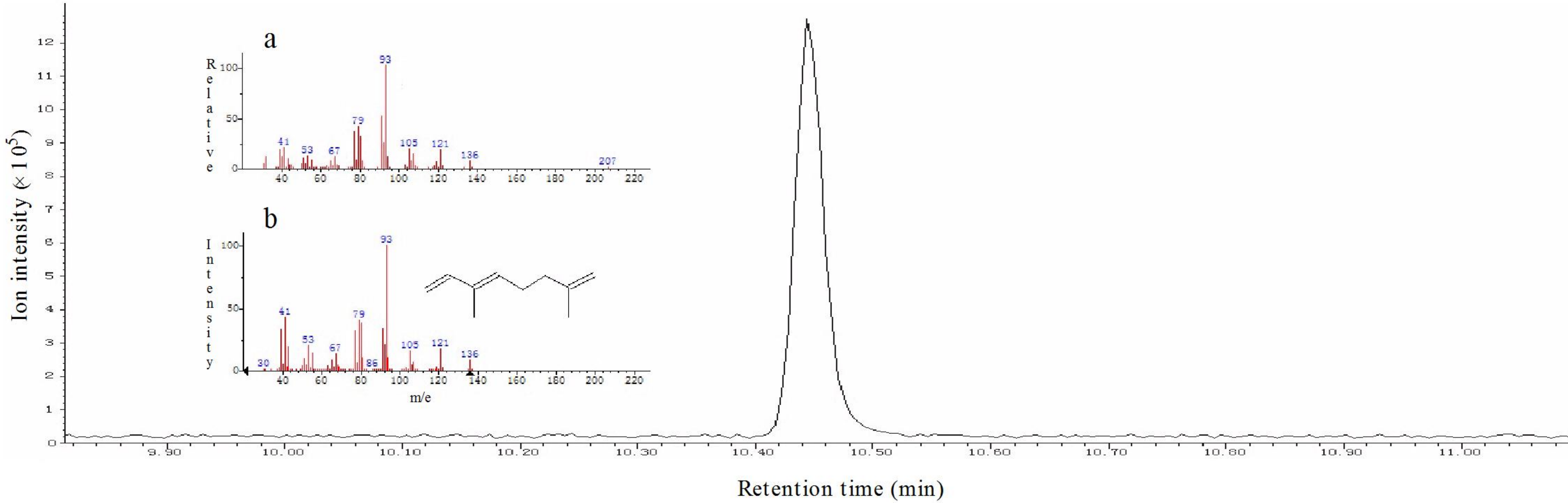

Figure S1. GC-MS analysis of E-*β*-ocimene from 2-day old starved larvae. The mass spectrum of this peak identified as E-*β*-ocimene (a) are shown together with that of the respective of authentic standards (b) from the NIST32I database.

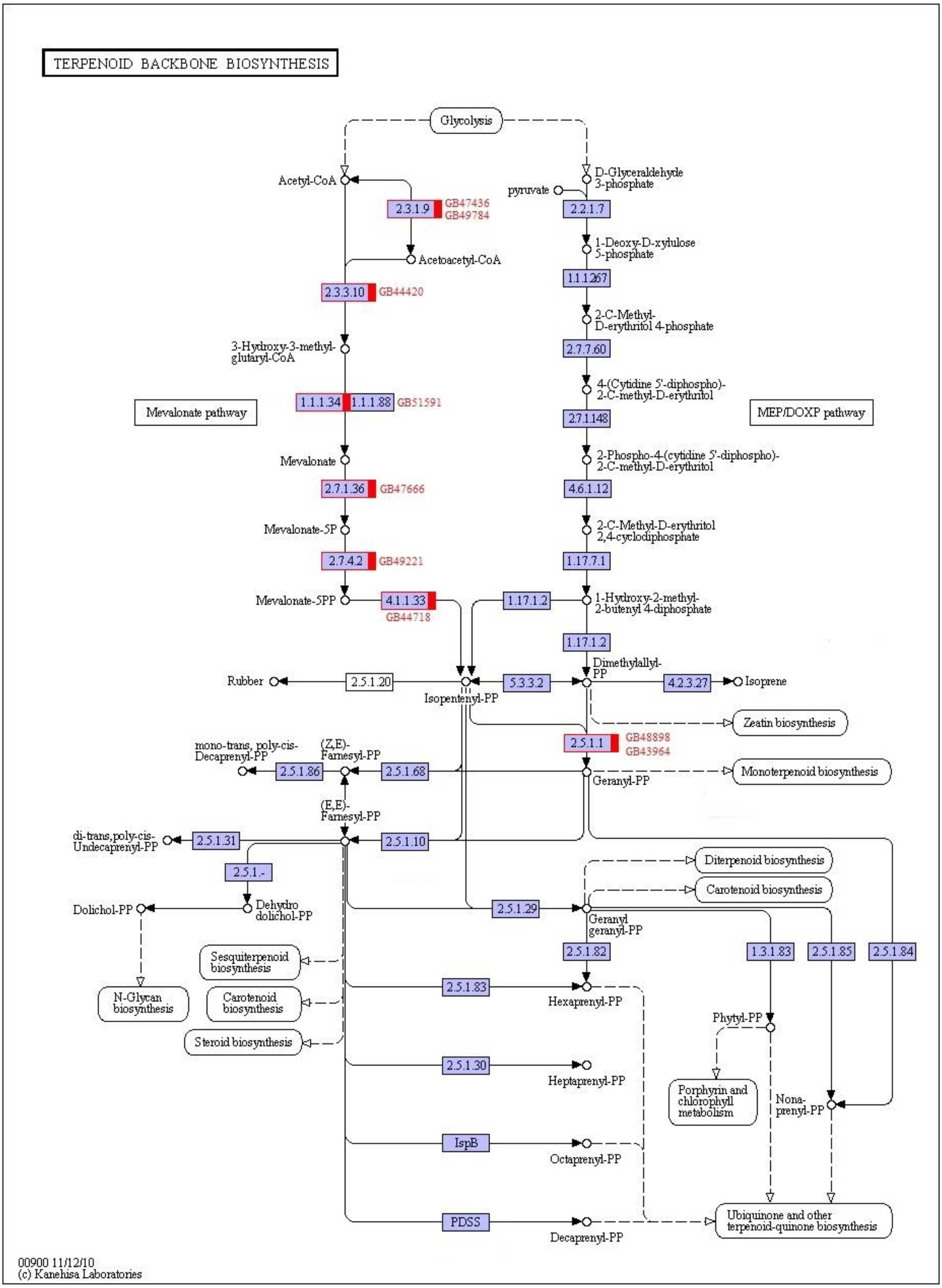

Figure S2. Biosynthetic pathways of E-β-ocimene in KEGG database. The Honey bee E-β-ocimene biosynthetic pathway and its genes are marked in (red).
